# Supplementary material for: A UMLS-based spell checker for natural language processing in vaccine safety
Source: BMC Med Inform Decis Mak. 2007 Feb 12;7:3. doi: 10.1186/1472-6947-7-3 (PMC1805499; doi:10.1186/1472-6947-7-3)
Supplement: Additional file 13 — RAP application directory. Sets up directory for RAP (RDF application for PHP) source code [file 1472-6947-7-3-S13.gz › rap/doc/phpdoc/__filesource/fsource_resModel__resModelResIterator.php.html]

xml version="1.0" encoding="iso-8859-1"?


File Source for ResIterator.php


# Source for file ResIterator.php

Documentation is available at ResIterator.php

```
1. <?php
2. /**
3. * ----------------------------------------------------------------------------------
4. * Class: ResIterator
5. * ----------------------------------------------------------------------------------
6. *
7. * @package     resModel
8. */


11. /**
12. * Implementation of a resource iterator.
13. *
14. * This Iterator should be used in a for-loop like:
15. * $it = $ontClass->listInstances();
16. * for ($it->rewind(); $it->valid(); $it->next())
17. * {
18. *    $currentResource=$it->current();
19. * };
20. *
21. * <BR><BR>History:
22. * <LI>10-07-2004                : First version of this class.</LI>
23. *
24. * @version  V0.9.1
25. * @author Daniel Westphal <mail at d-westphal dot de>
26. *
27. *
28. * @package     resModel
29. * @access    public
30. ***/
31. class ResIterator
32. {
33. /**
34. * Holds a reference to the assoiated ResModel / OntModel
35. * @var        object Model
36. * @access    private
37. */
38. var $associatedModel;

40. /**
41. * The current position
42. * @var        integer
43. * @access    private
44. */
45. var $key;

47. /**
48. * If the current resource is valid
49. * @var        boolean
50. * @access    private
51. */
52. var $valid;

54. /**
55. * The current resource
56. * @var obejct ResResource
57. * @access    private
58. */
59. var $currentResource;

61. /**
62. * The subject to search for.
63. * @var        object ResResource
64. * @access    private
65. */
66. var $searchSubject;

68. /**
69. * The predicate to search for.
70. * @var        object ResResource
71. * @access    private
72. */
73. var $searchPredicate;

75. /**
76. * The object to search for.
77. * @var        object ResResource
78. * @access    private
79. */
80. var $searchObject;

82. /**
83. * If the resource, we're intrested in is the subject (s), predicate(p),
84. * or object (o) of the found statements
85. *
86. * @var        string
87. * @access    private
88. */
89. var $getSPO;

91. /**
92. * Defines the type of resource, we'd like to receive.
93. *
94. * @var        string
95. * @access    private
96. */
97. var $returnType;

99. /**
100. * If set, each resource will first be checked, if it's
101. * language fits.
102. *
103. * @var        string
104. * @access    private
105. */
106. var $findLiteralWithLang;


109. /**
110. * Constructor.
111. *
112. * $subject, $predicate, and $object are used like inf find().
113. * $getSPO supports the strings 's', 'p', and 'o' to return
114. * either the subject, predicate, or object of the result statements.
115. * $returnType supports the strings 'ResProperty', 'ResLiteral',
116. * 'OntProperty', 'OntClass', and 'Individual' and returns the resources
117. * as the matching type.
118. *
119. * @param object ResResource  $subject
120. * @param object ResResource  $predicate
121. * @param object ResResource  $object
122. * @param string                 $getSPO
123. * @param object ResModel     $associatedModel
124. * @param string                 $returnType
125. * @access    public
126. */
127. function ResIterator($subject,$predicate,$object,$getSPO,& $associatedModel,$returnType = false)
128. {
129. $this->searchSubject =& $subject;
130. $this->searchPredicate =& $predicate;
131. $this->searchObject =& $object;
132. $this->getSPO = $getSPO;
133. $this->returnType = $returnType;
134. $this->associatedModel =& $associatedModel;
135. $this->findLiteralWithLang = false;
136. }

138. /**
139. * Resets iterator list to start
140. *
141. * @access    public
142. */
143. function rewind()
144. {
145. $this->key = -1;
146. $this->next();
147. }

149. /**
150. * Says if there are additional items left in the list
151. *
152. * @return    boolean
153. * @access    public
154. */
155. function valid()
156. {
157. return $this->valid;
158. }

160. /**
161. * Moves Iterator to the next item in the list
162. *
163. * @access    public
164. */
165. function next()
166. {
167. $this->key++;
168. $this->valid=($this->_getNextResource());
169. }

171. /**
172. * Returns the current item
173. *
174. * @return    mixed
175. * @access    public
176. */
177. function current()
178. {
179. return $this->currentResource;
180. }

182. /**
183. * Returns the next Resource (subject, predicate,
184. * or object of the next matching statement).
185. *
186. * @return    object resResource
187. * @access    private
188. */
189. function _getNextResource()
190. {
191. if ($this->findLiteralWithLang)
192. {
193. do
194. {
195. $nextStatement = $this->associatedModel->findFirstMatchingStatement($this->searchSubject,$this->searchPredicate,$this->searchObject,$this->key);
196. if ($nextStatement === null)
197. return false;

199. $object = $nextStatement->getObject();
200. if ($object->getLanguage() != $this->findLiteralWithLang)
201. {
202. $hasCorrectLanguage=false;
203. $this->key++;
204. } else
205. {
206. $hasCorrectLanguage=true;
207. }

209. } while (!$hasCorrectLanguage);
210. } else
211. {
212. $nextStatement = $this->associatedModel->findFirstMatchingStatement($this->searchSubject,$this->searchPredicate,$this->searchObject,$this->key);
213. }
214. if ($nextStatement === null)
215. return false;

217. switch ($this->getSPO)
218. {
219. case 's':
220. $this->currentResource = $this->_getResourceAs($nextStatement->getSubject());
221. break;

223. case 'p':
224. $this->currentResource = $this->_getResourceAs($nextStatement->getPredicate());
225. break;

227. case 'o':
228. $this->currentResource = $this->_getResourceAs($nextStatement->getObject());
229. break;
230. }
231. return (true);
232. }

234. /**
235. * Returns the key of the current item
236. *
237. * @return    integer
238. * @access    public
239. */
240. function key()
241. {
242. return $this->key;
243. }

245. /**
246. * Sets that only Literals with the matching
247. * language should be returned
248. *
249. * @param    string
250. * @access    public
251. */
252. function setFindLiteralWithLang($language)
253. {
254. $this->findLiteralWithLang = $language;
255. }

257. /**
258. * Returns the $resource as an instance of the type
259. * specified in $this->returnType.
260. *
261. * @param    object ResResource
262. * @return    object ResResource
263. * @access    private
264. */
265. function _getResourceAs($resource)
266. {
267. if ($this->findLiteralWithLang && $resource->getLanguage() != $this->findLiteralWithLang)
268. $this->_getNextResource();

270. if($this->returnType)
271. switch ($this->returnType) {

273. case 'ResProperty':
274. return $this->associatedModel->createProperty($resource->getLabel());
275. break;

277. case 'ResLiteral':
278. $newLiteral = $this->associatedModel->createLiteral($resource->getLabel(),$resource->getLanguage());
279. $newLiteral->setDatatype($resource->getDatatype());
280. return $newLiteral;
281. break;

283. case 'OntProperty':
284. return $this->associatedModel->createOntProperty($resource->getLabel());
285. break;

287. case 'OntClass':
288. return $this->associatedModel->createOntClass($resource->getLabel());
289. break;

291. case 'Individual':
292. return $this->associatedModel->createIndividual($resource->getLabel());
293. break;
294. }
295. return $resource;
296. }
297. }
```

Documentation generated on Thu, 7 Jul 2005 13:43:33 +0200 by phpDocumentor 1.3.0RC3
